# Postoperative fractionated stereotactic radiotherapy for completely resected brain metastases with 25 Gy in five fractions: A single-center retrospective study

**DOI:** 10.1016/j.ctro.2026.101198

**Published:** 2026-05-26

**Authors:** Sebastian H. Maier, Alexander Nitschmann, Diana-Coralia Dehelean, Lea Horne, Cecile Baumgartner, Vasiliki Anagnostatou, Sylvia Garny, Daniel F. Fleischmann, Montserrat Pazos, Veit M. Stoecklein, Louisa von Baumgarten, Niklas Thon, Florian Ringel, Claus Belka, Stefanie Corradini, Sebastian N. Marschner, Maximilian Niyazi, Raphael Bodensohn, Stephan Schönecker

**Affiliations:** a Department of Radiation Oncology, LMU University Hospital, LMU Medizin, LMU Munich, Munich, Germany; b Bavarian Cancer Research Center (BZKF), Munich, Germany; c German Cancer Consortium (DKTK), Partner Site Munich, A Partnership between DKFZ and LMU University Hospital, Munich, Germany; d Department of Neurosurgery, LMU University Hospital, LMU Medizin, LMU Munich, Munich, Germany; e Department of Neurosurgery, University Medical Center Knappschaftskrankenhaus Bochum, Ruhr University Bochum (RUB), 44892 Bochum, Germany; f Department of Radiation Oncology, Universitätsklinikum Erlangen, Friedrich-Alexander Universität Erlangen-Nürnberg, Erlangen, Germany; g Bavarian Cancer Research Center (BZKF), Erlangen, Germany; h Department of Radiation Oncology, University Hospital Tübingen, Tübingen, Germany; i Center for Neuro-Oncology, Comprehensive Cancer Center Tübingen-Stuttgart, University Hospital Tübingen 72076 Tübingen, Germany; j German Cancer Consortium (DKTK), Partner Site Tübingen, A Partnership between DKFZ and University Hospital, Tübingen, Germany

**Keywords:** Brain metastases, Fractionated stereotactic radiosurgery, Resection cavity, Recurrence, Stereotactic radiotherapy, Surgery, Radiation necrosis

## Abstract

**Background and purpose:**

Brain metastases (BM) occur in up to 40% of patients with solid tumors. Following surgical resection, postoperative radiotherapy to the resection cavity is standard of care, yet optimal dose prescription and fractionation remain uncertain. This study evaluates clinical outcomes and toxicity of a standardized postoperative fractionated stereotactic radiotherapy (fSRT) regimen.

**Materials and methods:**

This retrospective single-center study included patients treated between 2017 and 2025 with postoperative fSRT for completely resected brain metastases. All patients received 25 Gy in five fractions, prescribed to the 80% isodose line. The planning target volume (PTV) was defined as the resection cavity plus a 2-mm margin. Primary endpoints were local control (LC) and symptomatic radiation necrosis. Secondary endpoints included overall survival (OS) and intracranial progression-free survival (iPFS). Survival outcomes were estimated using the Kaplan-Meier method, and Cox regression analyses were performed to identify prognostic factors.

**Results:**

A total of 159 patients and 181 resection cavities were analyzed. The median PTV was 22.3 cc, while the median clinical target volume (CTV) measured 13.8 cc. Median follow-up was 48 months. Local control rates at 12 and 48 months were 91.8% and 89.4%, respectively. Symptomatic radiation necrosis occurred in 3.2% of patients. Median OS was 31 months, and median iPFS was 20 months. In multivariable analysis, uncontrolled extracranial disease was the only independent predictor of inferior OS (hazard ratio 2.58, p < 0.001).

**Conclusion:**

Postoperative fSRT with 5 × 5 Gy prescribed to the 80% isodose line is safe, well-tolerated, and provides durable local control after surgical tumor removal. Additional evidence is eagerly anticipated from the ongoing phase 3 trials.

**Trial registration:**

The local ethical review committee of Ludwig-Maximilians-University Munich (application number: 24–0206) approved this study. The study has been officially registered on the German Clinical Trials Register (DRKS00034150) on December 20, 2024.

## Highlights

• Adjuvant postoperative fSRT with 5 × 5 Gy after complete tumor resection achieved excellent long-term local control.
• Symptomatic radiation necrosis was rare despite extended follow-up.
• Uncontrolled extracranial disease independently predicted worse survival.

## Introduction

Brain metastases (BM) represent the most prevalent intracranial tumors in adults, affecting 20–40% of all cancer patients [1], [2], [3]. Their incidence is rising as survival is increasing due to effective systemic treatment, with the most frequent primary cancers being lung, breast, melanoma, and renal cell carcinoma [2]. Effective management of BM requires a multidisciplinary approach that incorporates systemic therapies, surgical intervention, stereotactic radiosurgery (SRS), fractionated stereotactic radiotherapy (fSRT), and whole-brain radiation therapy (WBRT) [2], [3], [4]. Historically, postoperative WBRT has been the standard treatment due to its effectiveness in reducing the risk of local recurrence in the surgical bed and the incidence of new distant metastases [5]. However, WBRT is associated with significant neurocognitive side effects, which have prompted a shift towards more focal treatments such as SRS and fSRT [6], [7]. Adjuvant RT is necessary to increase postoperative local control [8]. Randomized trials have demonstrated that postoperative SRS shows no differences in overall survival compared to WBRT while minimizing neurocognitive decline [6], [8]. Nevertheless, the optimal fractionation and dose for adjuvant RT remain uncertain [4]. Single fraction SRS usually needs to reduce radiation doses to larger resection cavities, resulting in higher recurrence rates especially for target lesions exceeding a maximal diameter of 3 cm [4], [9], [10]. FSRT combines the precision of SRS with the biological benefits of fractionation [10] and has demonstrated excellent local tumor bed control in several retrospective studies [11], [12], [13], [14], [15], with 12-month control rates exceeding those of SRS (average 79% for SRS vs. 91% for fSRT) [16]. Fractionated regimens allow the delivery of biologically effective tumor doses while reducing toxicity to normal brain tissue by allowing sublethal damage to be repaired between fractions. This achieves a favorable therapeutic ratio by maintaining efficacy without increasing the risk of radionecrosis (RN) [4], [10], [14], [16], [17]. Hypofractionation using 5 fractions is a widely adopted approach, with retrospective analyses demonstrating favorable local control rates and manageable toxicity profiles [13], [14], [18], [19], [20], [21], [22]. The present study investigates the clinical efficacy and toxicity profile of fSRT using 25 Gy in five fractions prescribed to the 80% isodose line for postoperative brain metastases resection cavities.

## Materials and methods

### Study design

A retrospective observational cohort study was conducted at the University Hospital of Ludwig-Maximilians-University to investigate the effectiveness of radiation therapy after complete resection of brain metastases in the postoperative MRI. The study received approval from the local ethics committee (Nr. 24–0206) and was registered at the German Clinical Trials Register (DRKS00034150).

**Fig. 2:**
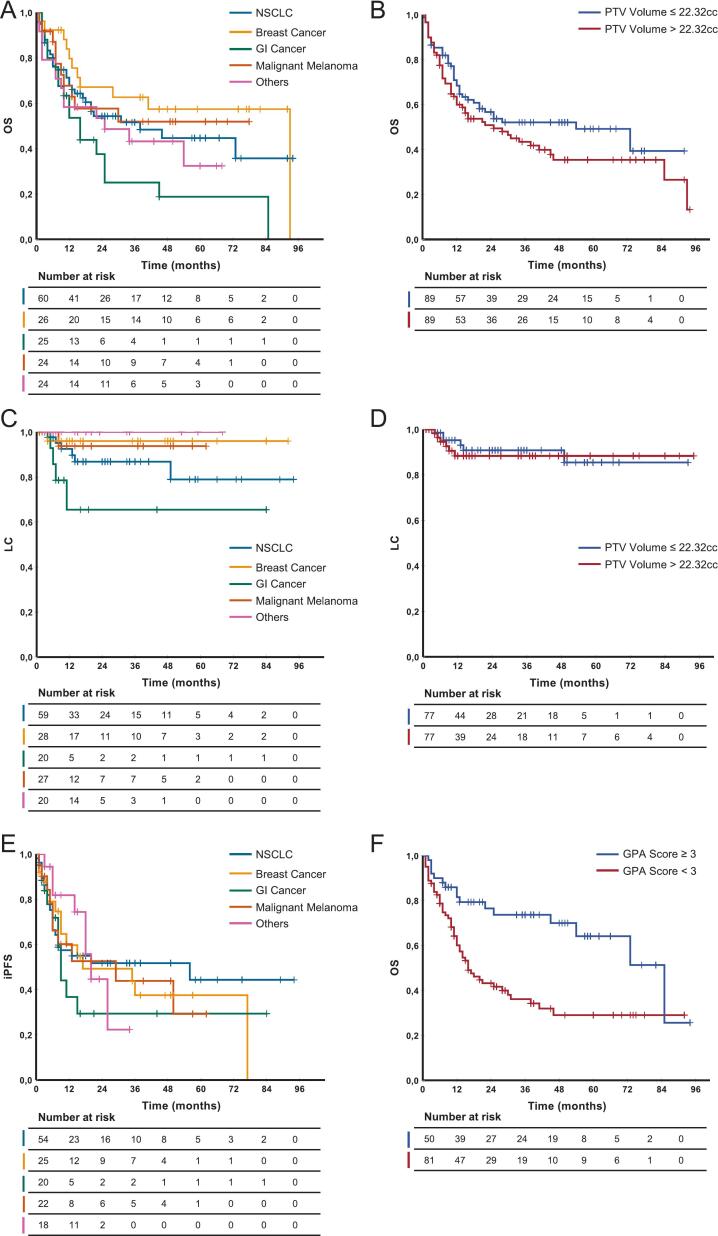
**Kaplan-Meier-Curves:** Kaplan-Meier estimates of OS, LC, and iPFS. **A**, **C**, and **E** show outcomes stratified by primary tumor entity, while **B**, **D** show outcomes stratified by median PTV size. **F** shows OS stratified by GPS Score. *LC = Local Control; iPFS = intracranial progression-free survival; OS = Overall Survival; PTV = planning target volume, GPA = Graded Prognostic Assessment.*

### Inclusion criteria

The inclusion criteria consisted of consecutively treated adult patients who received radiation therapy targeting the resection cavity after surgical intervention between 2017 and 2025 who received 5 fractions of 5 Gy on consecutive working days, to a total of 25 Gy to the 80% isodose line without concurrent or prior WBRT. In accordance with our internal standard for the treatment of brain metastases, the investigated dosage was exclusively applied to patients with no evidence of residual tumor in early postoperative MRI (performed within 48–72 h of the resection). No restrictions were imposed with respect to the primary histology.

### Radiation treatment planning and dose delivery

Planning was based on CT (slice thickness 1 mm) in combination with a recent cMRI (contrast-enhanced 3D T1-weighted MPRAGE sequence, acquired within 7 days of RT), with a planning target volume (PTV) margin following Choi et al. of 2 mm added to the resection cavity, defined as the clinical target volume (CTV) [23]. The CTV did not routinely include the surgical tract; however, in cases of preoperative dural contact, it was extended along the bone flap beyond the preoperative tumor contact [4]. Distortion correction (Elements, Brainlab SE, Munich, Germany) was usually performed after registration of CT and MRI images. Treatment planning was performed using the Monaco® treatment planning system (Elekta AB, Stockholm, Sweden), employing non-coplanar volumetric modulated arc therapy (VMAT) to achieve a highly conformal dose distribution. Immobilization was achieved with individually manufactured thermoplastic stereotactic masks, either the Brainlab Cranial 4Pi Stereotactic Mask or the IT-V iCAST Head Micro Double mask system (ITV Innovative Technologie Völp e.U., Innsbruck, Austria) [24]. Image-guided positioning was verified prior to and during each fraction using the Brainlab ExacTrac Dynamic® System. Patients underwent irradiation with 6 megavoltage photons delivered by Elekta Versa HD linear accelerators. Dosimetric constraints are provided in Table 2.

### Data Collection and Follow-Up

Clinical data were retrieved from medical records. The variables included patient demographics such as sex, age and Karnofsky performance score (KPS) on the first day of fSRT, fSRT date, underlying diagnosis, number and location of brain metastases, primary cancer control, and presence of extracranial metastases. Extracranial disease status and primary cancer control were determined based on the latest medical examination prior to fSRT. Patients were followed up with MRIs about every 3 months after fSRT until the last follow-up appointment or earlier in cases of neurological deficits. The diagnosis of RN was made by a multidisciplinary committee in accordance with the DEGRO practical guideline [25] using MRI and FET-PET imaging or biopsy/surgery.

The Graded Prognostic Assessment (GPA) was obtained using the dedicated calculator, including the latest update on melanoma patients[26], [27], [28].^1^ Data concerning systemic therapy were collected from the internal pharmacy database whenever available. Any administration of systemic therapy within 14 days preceding or following fSRT was designated as simultaneous [29].

### Statistics

The study's primary endpoints were local control (LC) and incidence of RN. Secondary outcomes included overall survival (OS) and distant intracranial progression-free survival (iPFS), defined as the time from fSRT to any new intracranial progression outside the treated cavity. Time to each endpoint was measured from the first day of the first fSRT, while LC and RN of sequentially treated resection cavities were calculated from each RT. OS, LC, incidence of RN and iPFS were estimated using the Kaplan-Meier method, with RN and LC analyzed per resection cavity if a follow-up MRI was available. In cases where WBRT was performed due to new intracranial metastases, LC was censored at the time of the procedure. Median follow-up time was calculated using the reverse Kaplan-Meier method with indicator variables reversed. Volumetric changes between GTV_preOP and CTV_postOP were analyzed in cases with available data. SPSS statistical software (IBM Inc., v29.0, Armonk, New York, USA) and R Studio (v2025.09.2 + 418; R: R-4.5.2) were used for statistical analyses. Univariate and multivariable Cox proportional hazards regression analyses were performed to identify factors associated with OS and LC. Variables with p < 0.10 in univariate analysis as well as clinically relevant covariates were included in the multivariable model. Continuous variables were entered as continuous terms. Plan analysis was performed using ProKnow® (Elekta) [30]. The conformity index (CI) and gradient index (GI) were calculated for each plan to assess target conformity and dose fall-off as follows: $\mathrm{CI}=\frac{\mathrm{PIV}}{\mathrm{TV}}$; $\mathrm{GI}=\frac{\mathrm{PIVhalf}}{\mathrm{PIV}}$ *(PIV = prescription isodose volume; TV = Target volume; PIVhalf = isodose volume receiving half of the prescribed dose)* [31], [32]*.*

## Results

### Patient characteristics

The study included a total of 159 patients, consisting of 64 men (40%) and 95 women (60%) with a total of 181 resection cavities. The median age of all patients was 62 years (range 32–86 years). For 17 patients, no follow-up MRI was available. The median follow-up time was 48 months (CI 41.1–54.9 months). Table 1 presents the characteristics of patients and resection cavities at the time of fSRT. The most prevalent primary diagnosis was non-small cell lung cancer (NSCLC) (38%), followed by breast cancer (16%), gastrointestinal cancer (GI) (16%) and melanoma (15%). In 33% of cases (n = 53), BM were detected simultaneously with the initial diagnosis, defined as within 2 months. Active extracranial disease was evident in 63% of patients (n = 96) at the time of BM diagnosis. At the time of RT, 86% of patients (n = 139) had a KPS of ≥ 70%. The median interval between surgery and initiation of RT was 28 days (range: 9–109 days, 95% CI: 26.6–31.6). One outlier (109 days) was due to postoperative complications, including acquiring COVID-19. Concurrent systemic therapy was administered in 41 patients (26%). Regarding the GPA score, 15 patients (9%) had a score of 0–1, 44 (28%) had a score of 1.5–2, 48 (30%) had a score of 2.5–3, and 24 (15%) had a score of 3.5–4.

**Table 1:** Patient characteristics and treatment parameters.

	n	%
Age (in years)		
Mean	62.4	
Median	62	
Range	32–86	
Sex		
Female	95	60%
Male	64	40%
Target Volumes	181	
Primary tumor		
NSCLC	60	38%
Breast cancer	26	16%
Gastrointestinal cancer	25	16%
Malignant melanoma	24	15%
Others	24	15%
Time from first diagnosis of primary tumor to first diagnosis of brain metastases		
Synchronous BM (≤2 months)	53	33%
Metachronous BM (>2 Months)	106	67%
Time from resection to fSRT (days)		
Mean	29.6	
Median	28	
Range	9–109	
95% CI	27.6–31.6	
KPS		
100	10	6%
90	48	30%
80	48	30%
70	33	20%
<70	16	10%
n.a.	7	4%
Number of lesions at time of fSRT		
1	110	69%
≥2	49	31%
GPA score		
0–1	15	9%
1.5–2	44	28%
2.5–3	48	30%
3.5–4	24	15%
Extracranial control at time of fSRT		
Yes	56	37%
No	97	63%
Concurrent systemic therapy	41	26%
Carboplatin/Pemetrexed/Pembrolizumab	6	4%
Ipilimumab/Nivolumab	6	4%
Pembrolizumab monotherapy	5	3%
FOLFOX	2	1%
Nivolumab mono	2	1%
Pertuzumab/Trastuzumab	2	1%
Trastuzumab mono	2	1%
Others	16	11%

*NSCLC = non-small-cell lung carcinoma; BM = brain metastases; fSRT = fractionated stereotactic radiotherapy; KPS = Karnofsky Performance Status; GPA = graded prognostic assessment; FOLFOX: folinic acid (leucovorin, FOL), fluorouracil (5-FU, F), oxaliplatin (Eloxatin, OX)*.

### Radiotherapy

**Fig. 3:**
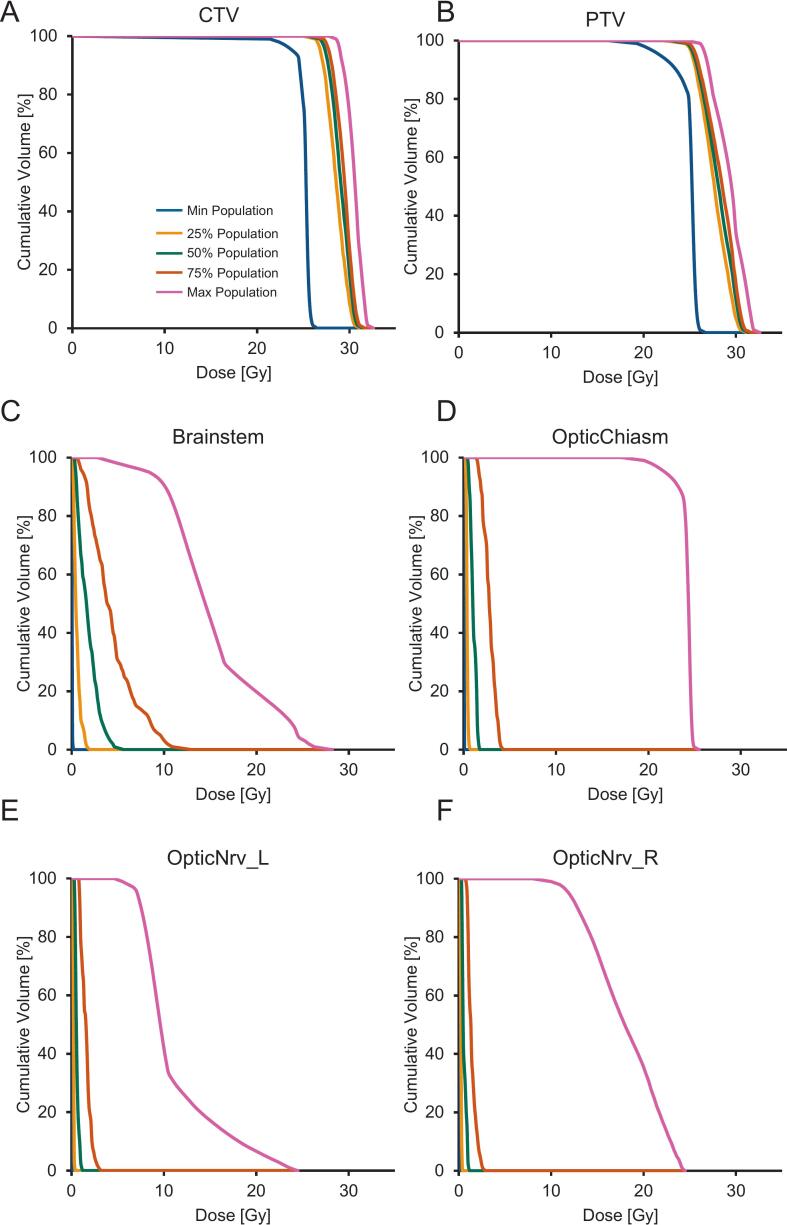
**Population DVH Analyses for A CTV, B PTV, C Brainstem, D OpticChiasm, E OpticNrv_L and F OptivNrv_R.** The min/max values and percentiles (25%, 50%, 75%) of the respective cohort are displayed.

Dosimetric parameters of the treated resection cavities are summarized in Table 2. The median PTV volume was 22.3 cc (range: 2.2–116.9 cc), while the median CTV measured 13.8 cc (range: 0.7–84.4 cc, **Fig. S1**). Target coverage was highly consistent, with a median PTV D98% of 25.0 Gy. Median PTV D50%, D2%, and Dmax were 28.1 Gy, 30.6 Gy, and 31.4 Gy, respectively. The median CI was 1.11, median GI was 3.29. Normal brain exposure (brain-CTV) remained limited, with median V18Gy, V20Gy, V24Gy, and V25Gy values of 24.8 cc, 20.0 cc, 10.9 cc, and 8.4 cc, respectively. The corresponding dosimetric parameters specific to patients with RN are presented in **Table S1**. Doses to organs at risk were low overall. The corresponding population DVH curves are shown in Fig. 3.

**Table 2:** Dosimetric data for treated surgical cavities.

	Mean	Median	Min	Max	Constraints
PTV volume (cc)	25.05	22.32	2.21	116.89	
CTV volume (cc)	16.00	13.76	0.69	84.36	
PTV D98% (Gy)	25.01	25.01	20.08	26.35	≥ 25 Gy
PTV D50% (Gy)	28.04	28.12	25.25	29.52	
PTV D2% (Gy)	30.46	30.64	25.98	31.82	≤ 31.25 Gy
PTV Dmax (Gy)	31.27	31.39	26.71	32.59	
PTV CI*	1.11	1.09	0.73	1.53	≤ 1.2
GI 12.5 Gy to 25 Gy*	3.34	3.29	1.95	7.02	≤ 4.5
Brain-CTV Dmax (Gy)*	29.53	29.54	26.62	31.62	
Brain-CTV D0.03 cc (Gy)*	28.96	28.97	26.35	31.07	
Brain-CTV D50% (Gy)*	2.73	2.34	0.61	29.96	
Brain-CTV V18Gy (cc)*	28.51	24.81	4.43	94.94	
Brain-CTV V20Gy (cc)*	23.00	20.01	2.54	72.53	≤ 20 cc
Brain-CTV V24Gy (cc)*	12.31	10.93	0.30	34.18	
Brain-CTV V25Gy (cc)*	9.22	8.42	0.10	25.57	≤ 16 cc
Brain-CTV V30Gy (cc)*	0.03	0.00	0.00	1.95	
Brain-CTV D20cc (Gy)*	19.52	20.23	6.64	27.80	
Brainstem D0.03 cc (Gy)	8.14	5.26	0.13	27.47	< 27 Gy
Brainstem D1.00 cc (Gy)	6.55	4.13	0.10	24.65	< 26 Gy
OpticChiasm D0.03 cc (Gy)	3.37	1.59	0.08	24.83	< 25 Gy
OpticNrv_L D0.03 cc (Gy)	2.19	0.96	0.05	21.88	< 25 Gy
OpticNrv_R D0.03 cc (Gy)	2.17	0.95	0.05	23.82	< 25 Gy
Cochlea_L D50% (Gy)	3.08	1.49	0.09	21.50	< 25 Gy
Cochlea_L Dmax (Gy)	3.92	2.02	0.10	24.76	< 25 Gy
Cochlea_R D50% (Gy)	2.51	1.30	0.05	16.44	< 25 Gy
Cochlea_R Dmax (Gy)	3.45	1.70	0.07	21.26	< 25 Gy

*D98% = minimum dose covering 98%; D90% = minimum dose covering 90%; V18Gy = Volume covered by 18 Gy; D0.03 = dose covering 0.03 cc; CI = Conformality index; GI = Gradient Index*.

*Analyzed in patients with one target volume per plan.

**Fig. 4:**
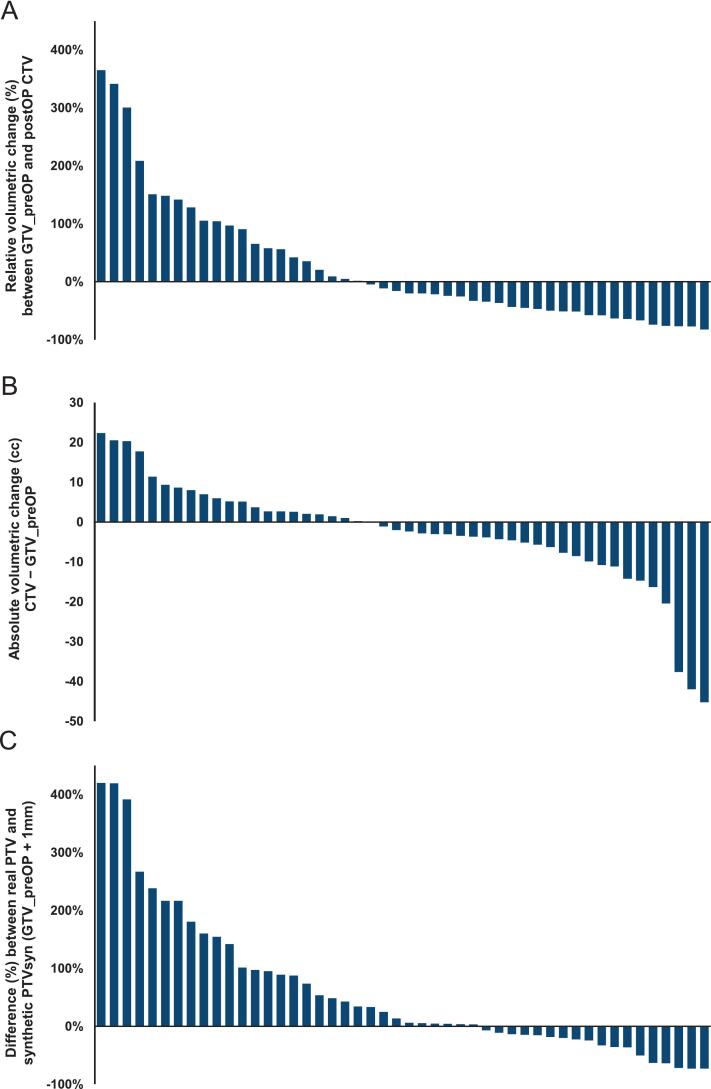
Waterfall plots showing A the relative volumetric change (%) between the preoperative gross tumor volume (GTV_preOP) and the postoperative resection cavity clinical target volume (CTV); B the absolute volumetric change (CTV − GTV_preOP, cc); and C the difference between the planning target volume (PTV) of the resection cavity and a synthetic stereotactic PTV (PTVsyn) generated by adding a 1-mm margin to the GTV_preOP. Bars are sorted by increasing change for individual patients. *GTV_preOP = preoperative gross tumor volume; CTV = clinical target volume; PTV = planning target volume; PTVsyn = synthetic stereotactic PTV.*

A sub-group analysis of patients (n = 48) for whom the planning data included a preoperative GTV (GTV_preOP) of the initial metastases revealed substantial interindividual variability in volumetric changes between the GTV_preOP and the postoperative CTV (Fig. 4A). While most cavities showed a reduction in size postoperatively, a considerable proportion demonstrated significant volumetric expansion. **Fig. S3** illustrates the volumetric change over time. A similar discrepancy was observed when comparing the postoperative PTV with a synthetic stereotactic PTV (PTVsyn) generated as GTV_preOP + 1 mm (Fig. 4B).

### Clinical outcome

**Fig. 1:**
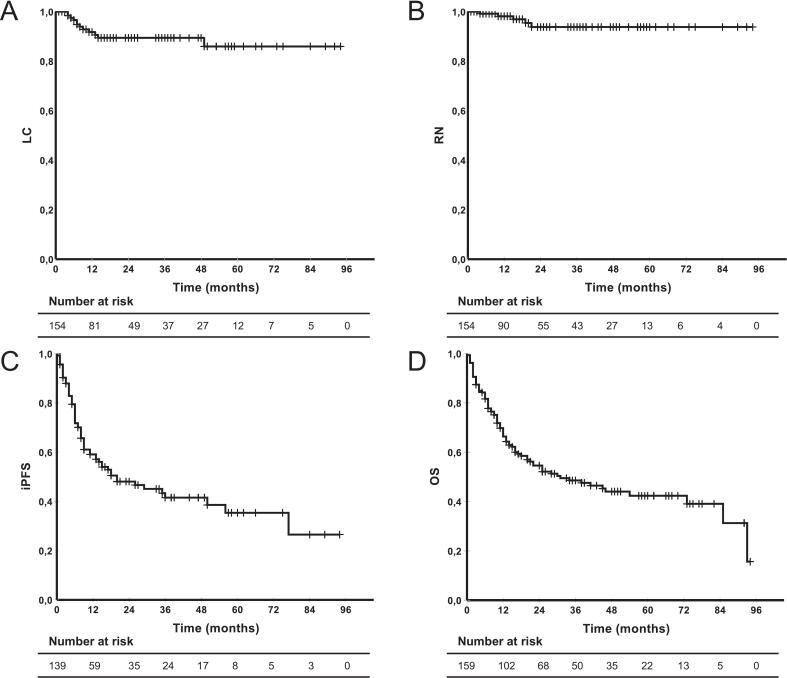
**Kaplan-Meier-Curves: A:** Local Control **B:** Radiation necrosis, **C:** Intracranial progression-free survival, **D:** Overall Survival. *LC = Local Control; RN = Radiation necrosis; iPFS = intracranial progression-free survival; OS = Overall Survival.*

Local control rates at 6, 12, and 48 months were 96.6%, 91.8%, and 89.4%, respectively (Fig. 1). Detailed information of patients suffering from local failure is provided in Table 3**A** including salvage therapy. Symptomatic RN occurred in 5 patients (3.2%) after a median of 15 months. Characteristics of patients who developed radiation necrosis are presented in Table 3**B**. **Fig. S2** shows the relationship between RN and PTV volume over time since RT. The median overall survival (OS) was 31 months (CI 13.9–48.1 months), although there were substantial differences between the individual tumor entities. NSCLC showed a median OS of 38 months (CI 6.9–69.1 months), breast cancer of 93 months (CI n/a), while GI tumors reached only 16 months (CI 9.6–22.3 months). The corresponding Kaplan-Meier analyses are shown in Fig. 2. The 12-month distant iPFS was 59.1%, while the median was 20 months (CI 4.2–35.8 months). 16 patients (10%) received WBRT during follow-up due to new brain metastases. In two cases, treatment with bevacizumab [33], [34] was indicated and was carried out in one case.

**Table 3:** A: Characteristics of patients who developed local failure:

#	Age	Sex	Primary tumor	Systemic therapy*	GPA Score	Time to Progress	PTV [cc]	Salvage
1	79	m	Gastrointestinal cancer (Rectum)	n.a.	3.5	5 months	34.7	OP
2	64	m	Gastrointestinal cancer (Rectum)	none	3.5	6 months	30.0	OP
3	66	f	NSCLC	none	2.5	49 months	12.9	Re-RT 1x20 Gy
4	71	f	NSCLC	none	0.5	4 months	33.6	None
5	81	f	Melanoma	Nivolumab	2.0	8 months	60.7	Re-RT 1x18 Gy
6	57	f	Breast cancer	Paclitaxel	2.0	4 months	14.4	None
7	59	m	NSCLC	none	3.5	9 months	46.9	OP
8	72	m	Gastrointestinal cancer (Colon)	Panitumu-mab	0.5	11 months	56.4	C2-WBRT
9	54	m	NSCLC	Pembrolizu-mab	2.5	14 months	11.8	OP, BSC
10	78	f	NSCLC	Alectinib	2.0	13 months	15.3	Re-RT 5x6 Gy
11	57	f	Gastrointestinal cancer (Colon)	none	3.5	7 months	5.3	Neoadjuvante Re-RT 5x5 Gy, OP
12	60	m	NSCLC	none	3.5	7 months	16.2	OP, Re-RT 3x5 Gy (stopped), Re-OP, WBRT + SIB + SIP

*GPA = graded prognostic assessment; PTV = Planning Target Volume; NSCLC = non-small cell lung cancer; OP = surgery; Re-RT = reirradiation; WBRT = whole brain radiotherapy; SIB = simultaneous integrated boost; SIP = simultaneous integrated protection; Age at fSRT; Systemic therapy at local progress*.

**Table 3:** B: Characteristics of patients who developed radiation necrosis:

#	Age	Sex	Primary tumor	Diagnostic	Time to RN	PTV [cc]	Therapy
1	47	f	Breast cancer	MRI + FET-PET	10 months	47.9	Bevacizumab
2	67	m	Gastrointestinal cancer (Sigma)	MRI	19 months	11.4	Bevacizumab indicated, not administered
3	48	f	Breast cancer	MRI	4 months	93.8	Dexamethasone
4	57	f	NSCLC	FET-PET, OP	15 months	32.8	OP, histologically RN
5	58	f	NSCLC	MRI, seizure	21 months	9.9	Dexamethasone, Levetiracetam

*PTV = Planning Target Volume; NSCLC = non-small cell lung cancer; OP = surgery; RN = radiation necrosis; Re-RT = reirradiation; Age at fSRT*.

In univariable analyses (Table 4), female sex showed a trend toward improved overall survival compared with male sex (HR 0.65, 95% CI 0.42–1.01, p = 0.054), which is best explained by the relatively favorable survival of patients with breast cancer. Increasing age (continuous) was associated with higher mortality (HR 1.03 per year, 95% CI 1.01–1.05, p = 0.016), reflecting a 3% increase in the hazard of death per additional year. Primary tumor site showed a trend toward worse survival for GI compared with NSCLC (HR 1.76, 95% CI 0.98–3.18, p = 0.059), whereas breast cancer, melanoma, and other tumors were not significant. The presence of uncontrolled extracranial disease was strongly associated with worse survival (HR 2.46, 95% CI 1.46–4.15, p < 0.001). Patients were stratified according to the median PTV volume (22.3 cc). No statistically significant difference in OS (HR 1.33, 95% CI 0.88–2.01, p = 0.174 or LC (HR 1.19, 95% CI 0.38–3.70, p = 0.761) was observed between smaller and larger target volumes. Higher GPA scores showed improved survival (HR 0.68, 95% CI 0.53–0.88, p = 0.003; GPA 3–4 vs 0–2.5; HR 0.38, 95% CI 0.22–0.67, p < 0.001). In multivariable analyses adjusting for sex, age, primary tumor site, and uncontrolled extracranial disease, only uncontrolled extracranial disease remained a predictor of worse survival (HR 2.58, 95% CI 1.50–4.44, p < 0.001). Other variables were not independently significant. Univariate Cox regression analyses did not identify any clinical or treatment-related factor significantly associated with LC (Table 5). Age showed a borderline association with LC when analyzed as a continuous variable (p = 0.052), while sex, performance status, primary tumor entity, systemic therapy, time from surgery to RT, and GPA were not significantly associated with local control. Although not statistically significant, GI primaries showed a trend toward inferior local control compared with other tumor entities, which was also reflected by the Kaplan-Meier curve (Fig. 2 **A**). In an exploratory analysis comparing GI primaries versus all other histologies, GI tumors were associated with significantly inferior LC (HR 4.87, 95% CI 1.44–16.44, p = 0.011), although the CI remained wide due to the limited number of events. Although colon carcinomas accounted for only four cases, two of these developed local recurrence, contributing disproportionately to the 12 total local failures observed in the cohort. Due to the small number of local failure events and the absence of statistically significant predictors in univariate analysis, no multivariable analysis for local control was performed. In our subgroup analysis of patients with local failure (Table 3**A**), we observed no consistent association with systemic therapy, as the majority were not under systemic treatment at the time of recurrence.

**Table 4:** Cox Regression for Overall Survival.

**Variable**	**Univariate HR (95% CI)**	***p***		**Multivariable HR (95% CI)**	***p***
Sex (female vs male)	0.653 (0.423–1.007)	0.054		1.421 (0.837–2.411)	0.193
Age (continuous)	1.025 (1.005–1.046)	**0.016**		1.011 (0.990–1.034)	0.312
Age < 50 years	*Reference*				
Age 50–70 years	1.293 (0.612–2.733)	0.501			
Age > 70 years	1.994 (0.889–4.471)	0.094			
KPS (continuous)	0.986 (0.968–1.005)	0.147			
KPS (≥70%)	0.600 (0.307–1.171)	0.134			
Primary tumor					
NSCLC	*Reference*			*Reference*	
Breast Cancer	0.669 (0.335–1.340)	0.257		0.862 (0.397–1.870)	0.707
Gastrointestinal cancer	1.763 (0.979–3.175)	0.059		1.697 (0.910–3.163)	0.096
Malignant melanoma	0.892 (0.435–1.830)	0.756		0.870 (0.415–1.823)	0.712
Others	1.275 (0.674–2.411)	0.455		1.742 (0.843–3.598)	0.134
Systemic therapy (yes)	0.864 (0.522–1.431)	0.570			
Extracranial disease (yes)	2.461 (1.461–4.146)	**<0.001**		2.579 (1.497–4.443)	**<0.001**
Time OP-RT (continuous)	0.989 (0.971–1.006)	0.198			
Subsequent WBRT (yes)	1.294 (0.685–2.446)	0.427			
PTV volume	1.002 (0.991–1.013)	0.677			
PTV volume ≤ 22.32 cc	*Reference*				
PTV volume > 22.32 cc	1.330 (0.882–2.005)	0.174			
GPA (continuous)	0.678 (0.525–0.875)	**0.003**			
GPA (0–2.5)	*Reference*				
GPA (3–4)	0.383 (0.217–0.674)	**<0.001**			

*CI = confidence interval; HR = hazard ratio; KPS = Karnofsky Performance Status; NSCLC = non-small cell lung cancer; PTV = Planning Target Volume, 22.32 cc was used as the median split; GPA = Graded Prognostic Assessment*.

GPA was excluded from the multivariate analysis due to its nature as a composite variable.

**Table 5:** Cox Regression for Local Control.

**Variable**	**Univariate HR (95% CI)**	***p***
Sex (female vs male)	0.491 (0.158–1.528)	0.219
Age (continuous)	1.057 (1.000–1.117)	0.052
KPS (continuous)	0.992 (0.994–1.043)	0.763
KPS (≥70%)	0.958 (0.124–7.424)	0.976
Primary tumor		
NSCLC	*Reference*	
Breast Cancer	0.317 (0.038–2.634)	0.288
Gastrointestinal cancer	2.723 (0.755–9.822)	0.126
Malignant melanoma	0.387 (0.047–3.214)	0.379
Others	0.000	0.977
Systemic therapy (yes)	1.139 (0.342–3.787)	0.832
Time OP-RT (continuous)	0.965 (0.916–1.017)	0.184
PTV volume (continuous)	1.011 (0.984–1.037)	0.432
PTV volume ≤ 22.32 cc	*Reference*	
PTV volume > 22.32 cc	1.192 (0.384–3.700)	0.761
GPA (continuous)	0.897 (0.481–1.673)	0.732
GPA (0–2.5)	*Reference*	
GPA (3–4)	0.705 (0.222–2.238)	0.553

*CI = confidence interval; HR = hazard ratio; KPS = Karnofsky Performance Status; GPA = Graded Prognostic Assessment; NSCLC = non-small cell lung cancer*.

## Discussion

In the present study, we analyzed clinical outcomes following adjuvant fSRT with 25 Gy in five fractions prescribed to the 80% isodose line after complete resection of BM, highlighting LC, OS and iPFS. Excellent long-term LC with a low incidence of symptomatic RN could be shown despite a BED10 under 40 Gy [4]. LC at 12 and 48 months (91.8% and 89.4%, respectively) and a 3.2% rate of symptomatic RN support the safety and efficacy of this regimen in carefully selected patients with completely resected brain metastases.

Compared to the recent study by Carpenter et al., which analyzed outcomes of 445 patients (53% resected, 47% intact) treated with 5-fraction SRS at 5–6 Gy per fraction, our cohort showed improved LC. In that study, the cumulative incidence of local progression at 12 and 18 months was 13.6% and 14.5% amounting to a local control rate of 86.4% and 85.5%, respectively. Moreover, the iPFS of 20 months in our series compares favorably to the 12-month distant brain progression (DBP) rate of 46.1% reported by Carpenter et al. While DBP and iPFS are not fully equivalent endpoints, these data collectively suggest a meaningful prolongation of intracranial disease control in our population.

Our findings also align with those from other large retrospective series using similar dose and fractionation. Abuodeh et al. reported 1- and 2-year LC rates of 88.8% and 83.9%, respectively, using 25 Gy in 5 fractions (prescribed with 95% minimum coverage of PTV), with a 3% RN rate. These outcomes are remarkably consistent with ours, reinforcing the reproducibility and robustness of this regimen across institutions. However, the extended median follow-up in our study of 48 months compared to 9.5 months adds important evidence regarding the long-term durability of LC. Furthermore, our median iPFS of 20 months and 12 months rate of 59.1% again align well with the 1-year distant brain control rate of 53.9% reported in the Abuodeh cohort. Notably, the observed median OS in our study of 31 months (12/24 months OS rates of 66.4% and 54.6%) exceeds that reported in the Carpenter (12.8 months) cohort and aligns with the Abuodeh cohort (12/24 months OS rates of 73.1% and 62.2%). While performance status was broadly comparable, the outcomes in our cohort may reflect both favorable patient selection and a more contemporary treatment period (2017–2018) [21]. During this time, the widespread integration of systemic therapies with CNS activity – including targeted therapies and immune checkpoint inhibitors – particularly for NSCLC, melanoma and breast cancer, likely contributed to improved extracranial and intracranial control and ultimately prolonged survival [16], [35].

In the present study, only 10% of patients required subsequent WBRT, indicating the efficacy of initial fSRT in sparing or delaying WBRT. Concurrently, this development signifies a transition from palliative WBRT to a more targeted, individualized multimodal tumor therapy approach, underscoring the evolution of cancer treatment strategies [3], [7], [35].

The findings of this study also demonstrate a remarkably low adverse event profile, with symptomatic RN observed in only 3.2% of patients, which is significantly lower compared to other recently reported series. For instance, Hahnemann et al. reported that, out of 105 patients treated with fSRT (5x7Gy 70% isodose, 1-year-LC 98.6%), 20 experienced symptomatic radiation-induced contrast enhancement in the cavity, with 18 requiring intervention such as corticosteroids or bevacizumab [14]. Carpenter et al. reported an 11% rate of grade 2 or higher RN within patients with resected BM [21]. The low incidence of symptomatic RN in our cohort underscores the safety profile of the 5x5Gy regimen, particularly when considering the therapeutic window between efficacy and adverse effects.

The timing of postoperative RT is crucial for optimizing clinical outcome [9]. Research shows that the cavity volume can decrease by up to 43% within the first three to four weeks [4]. Delays beyond this period may lead to the spread of microscopic disease, which is undetectable on imaging, resulting in increased local failure [4], [36]. The median interval of 28 days observed in this study aligns with the consensus for treatment within 4 weeks post-surgery [4].

Recent studies have indicated that neoadjuvant RT may offer certain advantages by decreasing the likelihood of postoperative leptomeningeal spread and reducing the risk of RN [37], [38], [39], [40]. There is evidence to suggest that fSRT may be a preferable option for neoadjuvant RT (1x15Gy vs. 3x8Gy) [40]. However, the implementation of neoadjuvant RT presents logistical challenges and may not be feasible in cases of urgent surgical intervention. And, as shown in our analysis, the primary target volumes are not automatically smaller than in the postoperative situation (Fig. 4). Current prospective phase III trials (NCT05438212/NRG-BN012; NCT04474925; PREOP-2/NCT05124236) are investigating the comparative efficacy of neoadjuvant versus adjuvant radiosurgery [3], [40], [41], [42].

Notwithstanding the encouraging results of adjuvant fSRT, the most effective treatment strategy remains a subject of debate. A critical evaluation of our study reveals both notable strengths and important limitations. Chief among the strengths is the large and homogeneous patient cohort – one of the largest treated with a consistent 5x5Gy fSRT protocol after complete resection with no residual tumor – allowing for meaningful conclusions regarding efficacy and safety. Nevertheless, the retrospective nature of the study introduces inherent limitations, including potential selection bias and variable follow-up. Operability is often closely associated with more favorable prognostic characteristics and overall survival. While the excellent LC rates are promising, they may partially reflect underreporting or loss to follow-up. The requirement of a postoperative MRI confirming the absence of residual tumor ensured a well-defined and homogeneous cohort. In this setting, a relative dose de-escalation compared with commonly used higher-dose regimens [4] could be safely applied. This approach differs from situations in which residual disease is present or cannot be reliably excluded, where dose intensification may be warranted. Incomplete documentation of adverse events further limits the assessment. The forthcoming Alliance A071801 trial (NCT04114981) seeks to provide more definitive guidance by comparing SRS and fSRT in patients with resected brain metastases and additional unresected lesions [16], [43]. The results of this randomized trial are awaited with interest to further clarify the long-term benefits and safety profiles of these regimens.

## Conclusion

In conclusion, the present study demonstrates that a postoperative fractionated RT regimen with 25 Gy in five fractions to the 80% isodose is feasible and well-tolerated and offers excellent local control in carefully selected patients after complete tumor resection. The low rate of RN is particularly notable. Additional evidence is eagerly anticipated from the ongoing phase 3 trials.

**Ethics approval**

Approval for this study was granted by the local ethics committee of Ludwig-Maximilians-University Munich (application number: 24–0206). The study has been officially registered with the German Clinical Trials Register (DRKS00034150).

**Consent for publication**

Not applicable due to the retrospective nature of the analysis and the use of non-identifiable data.

**Availability of data and materials**.

The datasets used and analyzed during the current study are available from the corresponding author on reasonable request.

## CRediT authorship contribution statement

**Sebastian H. Maier:** Formal analysis, Investigation, Writing - Original Draft, Visualization. **Alexander Nitschmann:** Writing – review & editing, Investigation. **Diana-Coralia Dehelean:** Writing – review & editing, Investigation. **Lea Horne:** Writing – review & editing, Investigation. **Cecile Baumgartner:** Writing – review & editing, Investigation. **Vasiliki Anagnostatou:** Writing – review & editing, Visualization, Software, Investigation. **Sylvia Garny:** Writing – review & editing, Software, Investigation. **Daniel F. Fleischmann:** Writing – review & editing, Investigation. **Montserrat Pazos:** Writing – review & editing, Investigation. **Veit M. Stoecklein:** Writing – review & editing, Investigation. **Louisa von Baumgarten:** Writing – review & editing, Investigation. **Niklas Thon:** Writing – review & editing, Investigation. **Florian Ringel:** Writing – review & editing, Investigation. **Claus Belka:** Supervision, Resources. **Stefanie Corradini:** Writing – review & editing, Methodology, Conceptualization. **Sebastian N. Marschner:** Writing – review & editing, Investigation. **Maximilian Niyazi:** Writing – review & editing, Validation, Supervision, Resources, Project administration, Methodology, Funding acquisition, Conceptualization. **Raphael Bodensohn:** Writing – review & editing, Validation, Methodology, Investigation, Conceptualization. **Stephan Schönecker:** Writing – review & editing, Validation, Supervision, Software, Methodology, Investigation, Formal analysis, Conceptualization.

## Funding

This project has received funding from the Bavarian Cancer Research Center (BZKF).

## Declaration of competing interest

The authors declare that they have no known competing financial interests or personal relationships that could have appeared to influence the work reported in this paper.

## Appendix A Supplementary data

Supplementary Data 1 Figure S1: Distribution of clinical target volume (CTV) and planning target volume (PTV) in the study cohort. Violin plots display the full distribution with median and interquartile range. CTV = clinical target volume; PTV = planning target volume Figure S2: Analysis of patients with radionecrosis (RN). The size of the irradiated PTV was plotted against the time between RT and RN. RN = radionecrosis; PTV = planning target volume; RT = radiotherapy Volume reduction of the resection cavity (CTV-GTVpreOP) plotted against the time between surgery and the start of fSRT. CTV = clinical target volume; GTVpreOP = gross tumor volume of unresected brain metastases; fSRT = fractionated stereotactic radiotherapy.

Supplementary Data 2 Table S1:Dosimetric data for treated surgical cavities of patients with symptomatic RN (n=5)

## Footnotes

1 https://brainmetgpa.com/.
