# Supplementary material for: Postoperative fractionated stereotactic radiotherapy for completely resected brain metastases with 25 Gy in five fractions: A single-center retrospective study
Source: Clin Transl Radiat Oncol. 2026 May 26;59:101198. doi: 10.1016/j.ctro.2026.101198 (PMC13241780; doi:10.1016/j.ctro.2026.101198)

# Supplement

## Table S1: Dosimetric data for treated surgical cavities of patients with symptomatic RN (n=5)

|  | Mean | Median | Min | Max |
| --- | --- | --- | --- | --- |
| PTV volume (cc) | 39.16 | 32.76 | 9.89 | 93.83 |
| CTV volume (cc) | 27.52 | 22.32 | 4.90 | 71.57 |
| PTV D98% (Gy) | 25.16 | 25.01 | 24.98 | 25.63 |
| PTV D50% (Gy) | 27.75 | 27.80 | 27.12 | 28.29 |
| PTV D2% (Gy) | 30.13 | 30.15 | 29.42 | 30.78 |
| PTV Dmax (Gy) | 31.16 | 31.42 | 30.23 | 31.56 |
| Brain-CTV Dmax (Gy)* | 29.27 | 29.40 | 28.40 | 30.60 |
| Brain-CTV D0.03cc (Gy)* | 28.92 | 28.91 | 28.09 | 30.31 |
| Brain-CTV D50% (Gy)* | 2.15 | 1.53 | 1.03 | 5.30 |
| Brain-CTV V18Gy (cc)* | 27.32 | 18.44 | 13.89 | 60.69 |
| Brain-CTV V20Gy (cc)* | 22.03 | 15.19 | 11.62 | 48.66 |
| Brain-CTV V24Gy (cc)* | 12.19 | 8.79 | 6.80 | 26.37 |
| Brain-CTV V25Gy (cc)* | 9.22 | 6.96 | 5.45 | 19.12 |
| Brain-CTV V30Gy (cc)* | 0.01 | 0.00 | 0.00 | 0.07 |
| Brain-CTV D20cc (Gy)* | 18.52 | 17.12 | 14.04 | 24.89 |
| *D98% = minimum dose covering 98%; D90% = minimum dose covering 90%; V18Gy = Volume covered by 18 Gy; D0.03 = dose covering 0.03cc.*  **Analyzed in patients with one target volume per plan, one patient had two more metastasis treated in a timely manner.* | | | | |

**Figure S1:** Distribution of clinical target volume (CTV) and planning target volume (PTV) in the study cohort. Violin plots display the full distribution with median and interquartile range.

*CTV = clinical target volume; PTV = planning target volume*


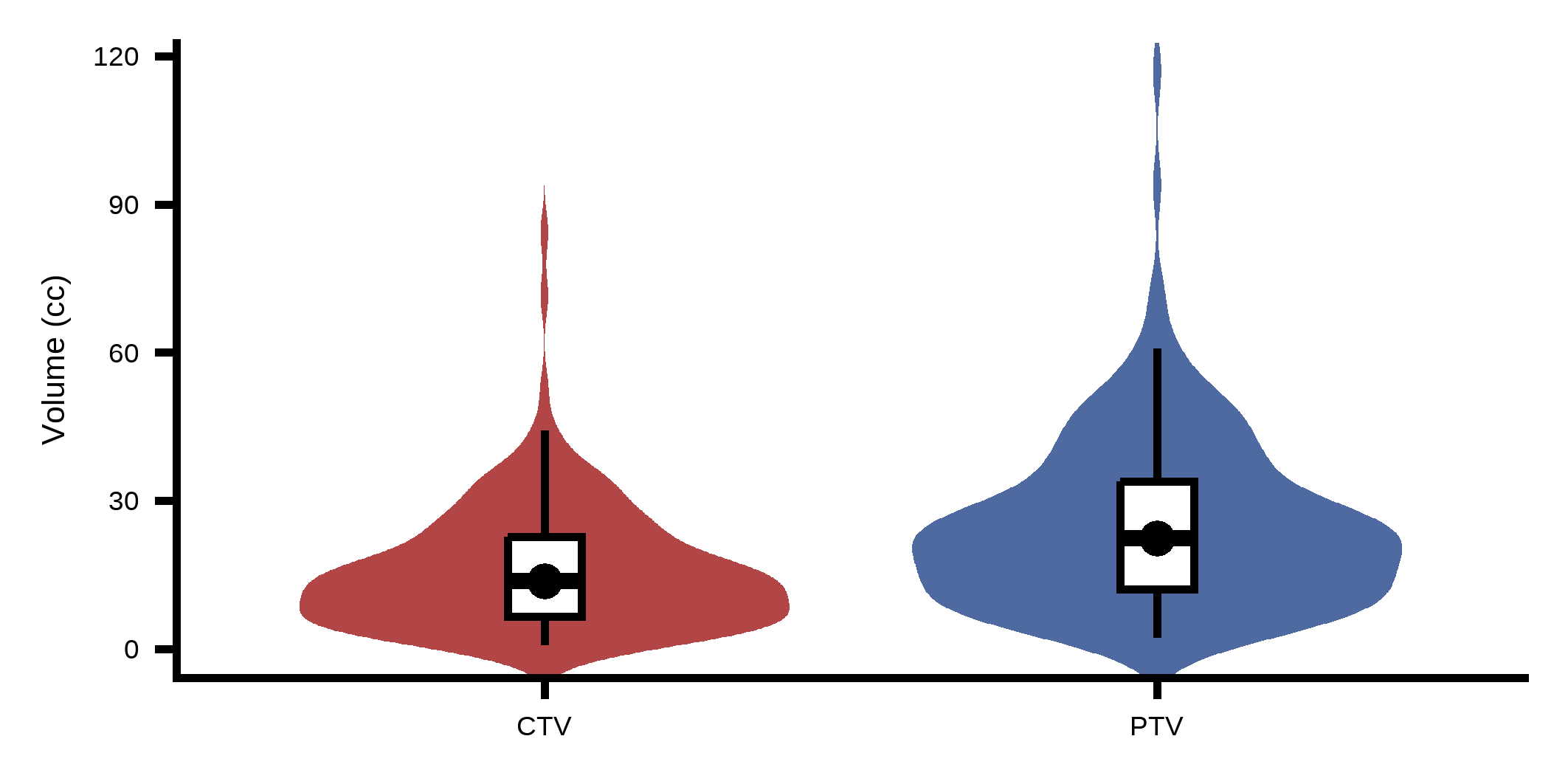


**Figure S2:** Analysis of patients with radionecrosis (RN). The size of the irradiated PTV was plotted against the time between RT and RN.

*RN = radionecrosis; PTV = planning target volume; RT = radiotherapy*


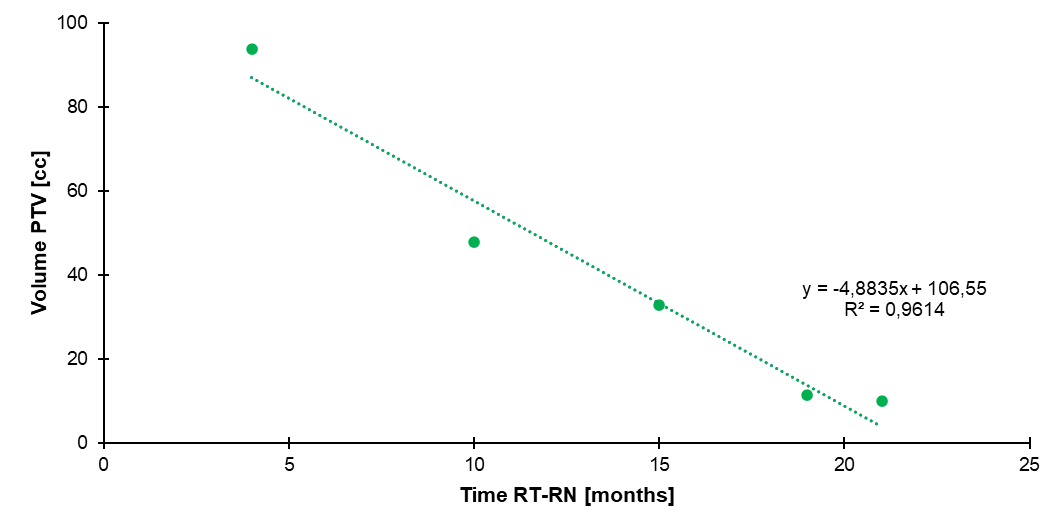


**Figure S3:** Volume reduction of the resection cavity (CTV-GTVpreOP) plotted against the time between surgery and the start of fSRT.

CTV = *clinical target volume; GTVpreOP = gross tumor volume of unresected brain metastases; fSRT = fractionated stereotactic radiotherapy.*


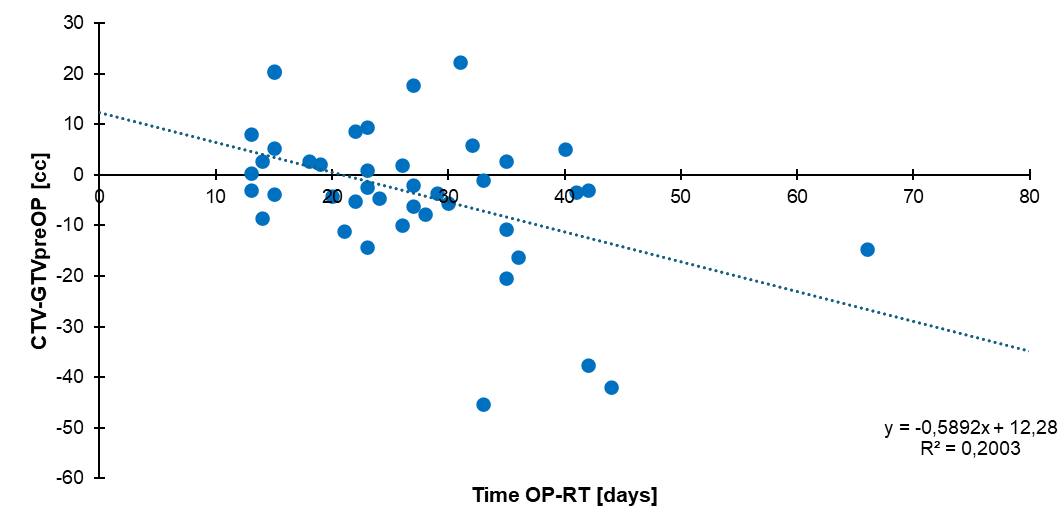

Supplement: Supplementary Data 1 — Figure S1: Distribution of clinical target volume (CTV) and planning target volume (PTV) in the study cohort. Violin plots display the full distribution with median and interquartile range. CTV = clinical target volume; PTV = planning target volume Figure S2: Analysis of patients with radionecrosis (RN). The size of the irradiated PTV was plotted against the time between RT and RN. RN = radionecrosis; PTV = planning target volume; RT = radiotherapy Volume reduction of the resection cavity (CTV-GTVpreOP) plotted against the time between surgery and the start of fSRT. CTV = clinical target volume; GTVpreOP = gross tumor volume of unresected brain metastases; fSRT = fractionated stereotactic radiotherapy. [file mmc1.docx]
