# Supplementary material for: Postoperative fractionated stereotactic radiotherapy for completely resected brain metastases with 25 Gy in five fractions: A single-center retrospective study
Source: Clin Transl Radiat Oncol. 2026 May 26;59:101198. doi: 10.1016/j.ctro.2026.101198 (PMC13241780; doi:10.1016/j.ctro.2026.101198)
Supplement: Supplementary Data 2 — Table S1:Dosimetric data for treated surgical cavities of patients with symptomatic RN (n=5) [file mmc2.pdf]

Table S1: Dosimetric data for treated surgical cavities of patients with symptomatic RN (n=5)

	Mean	Median	Min	Max
PTV volume (cc)	39.16	32.76	9.89	93.83
CTV volume (cc)	27.52	22.32	4.90	71.57
PTV D98% (Gy)	25.16	25.01	24.98	25.63
PTV D50% (Gy)	27.75	27.80	27.12	28.29
PTV D2% (Gy)	30.13	30.15	29.42	30.78
PTV Dmax (Gy)	31.16	31.42	30.23	31.56
Brain-CTV Dmax (Gy)*	29.27	29.40	28.40	30.60
Brain-CTV D0.03cc (Gy)*	28.92	28.91	28.09	30.31
Brain-CTV D50% (Gy)*	2.15	1.53	1.03	5.30
Brain-CTV V18Gy (cc)*	27.32	18.44	13.89	60.69
Brain-CTV V20Gy (cc)*	22.03	15.19	11.62	48.66
Brain-CTV V24Gy (cc)*	12.19	8.79	6.80	26.37
Brain-CTV V25Gy (cc)*	9.22	6.96	5.45	19.12
Brain-CTV V30Gy (cc)*	0.01	0.00	0.00	0.07
Brain-CTV D20cc (Gy)*	18.52	17.12	14.04	24.89

D98% = minimum dose covering 98%; D90% = minimum dose covering 90%; V18Gy = Volume covered by 18 Gy; D0.03 = dose covering 0.03cc.

**Analyzed in patients with one target volume per plan, one patients had two more metastasis treated in a timely manner.*
